# Prevalence and cumulative incidence of autism spectrum disorders and the patterns of co-occurring neurodevelopmental disorders in a total population sample of 5-year-old children

**DOI:** 10.1186/s13229-020-00342-5

**Published:** 2020-05-14

**Authors:** Manabu Saito, Tomoya Hirota, Yui Sakamoto, Masaki Adachi, Michio Takahashi, Ayako Osato-Kaneda, Young Shin Kim, Bennett Leventhal, Amy Shui, Sumi Kato, Kazuhiko Nakamura

**Affiliations:** 1 Department of Neuropsychiatry, Graduate School of Medicine, Hirosaki University, 5 Zaifu-cho, Hirosaki, Aomori, 036-8562 Japan; 2 Department of Psychiatry, Langley Porter Psychiatric Institute, University of California San Francisco, 401 Parnassus Avenue, San Francisco, CA 94143 USA; 3 Research Center for Child Mental Development, Graduate School of Medicine, Hirosaki University, 5 Zaifu-cho, Hirosaki, Aomori, 036-8562 Japan; 4 Department of Management and Law, Aomori Chuo Gakuin University, Aomori, 030-0132 Japan

**Keywords:** Prevalence, Cumulative incidence, Co-existing neurodevelopmental disorders, Autism spectrum disorder, A total population study

## Abstract

**Backgrounds:**

Whether there is a true increase in autism spectrum disorder (ASD) frequency or not remains unclear. Additionally, the rates of co-existing neurodevelopmental disorders (NDD) in a total population sample has not been fully examined before. Therefore, using a total population sample in Japan, we aimed to estimate the prevalence and cumulative incidence of autism spectrum disorder (ASD) annually, to determine whether there is a true increase in ASD prevalence by estimating the cumulative incidence of ASD annually, and to examine the rates of co-existing neurodevelopmental disorders (NDD).

**Method:**

In this cross-sectional sequential design study, all 5-year-old children in the catchment area underwent the screening annually from the year 2013–2016. Screen-positive children were invited to participate in a comprehensive assessment, including child and parent interview, behavioral observation, and cognitive and motor function testing. All cases were reviewed by a multidisciplinary research team.

**Results:**

Caregivers of 3954 children returned the screening, among which 559 children underwent the assessment with 87 children receiving an ASD diagnosis. Adjusted ASD prevalence was 3.22% (95% confidence interval (CI) 2.66–3.76%). The male to female ratio of the crude prevalence was 2.2:1. The cumulative incidence of ASD up to 5 years of age for the total study years was 1.31% (95% CI 1.00–1.62%). A generalized linear model revealed no significant linear trends in 5-year cumulative incidence over the study years. Only 11.5% of children had ASD alone; the remaining 88.5% were found to have at least one co-existing NDD.

**Limitations:**

Modest sample size for a total population study.

**Conclusions:**

Our findings demonstrate the stability of the 5-year cumulative incidence of ASD, implying no true rise in ASD incident cases over the 4-year study period in the study catchment area. High rates of co-existing NDDs reflect the importance of investigating broad developmental challenges in children with ASD.

## Introduction

The US Centers for Disease Control and Prevention has most recently reported an increase in the autism spectrum disorder (ASD) prevalence at 1 in 59 children [1] from that at 1 in 68 children in its previous report [2]. This trend is corroborated by epidemiological studies conducted in Asian countries [3, 4], consistently reporting that the ASD prevalence is higher than that previously reported in each country. In an epidemiological study targeting children aged 6–10 in China, the study findings revealed that ASD was under-detected in mainstream schools [3]. Another study that included five geographically diverse populations in India reported the ASD prevalence was 1.4% among children aged 6–9 years [4]. Furthermore, using a total population sample and rigorous case ascertainment, Kim et al. reported the prevalence estimate of ASDs was 2.64 % with 95% confidence interval (CI) being 1.91–3.37 in children in South Korea [5], which was higher than that previously reported. Most recently, a study led by the Health Resources and Service Administration using the 2016 National Survey of Children’s Health revealed that the ASD prevalence based on parent reports was 2.5% in the USA [6], supporting the findings from Kim et al. ’s study.

Despite a growing number of publications reporting the ASD prevalence, however, few studies have examined the ASD incidence, another measure of ASD frequency. The limited number of incidence studies is likely due to methodological challenges in estimating incidence [7]; more specifically, the difficulties in prospectively following up birth cohorts until the onset of ASD and in identifying the onset time of ASD, a common challenge in neurodevelopmental disorder. Instead, cumulative incidence measure can be used to overcome these challenges. For example, the cumulative incidence of childhood autism (based on the International Classification of Disease and Related Health Problems, 10th revision, [8]) up to 5 years of age was measured in a birth cohort in a population-based sample in Japan. In that study, the authors of the above-referenced study assumed that the cumulative incidence of ASD plateaus by age 5 because it is an early childhood-onset neurodevelopmental disorders (NDDs) [9]. The cumulative incidence is a valuable measure of the risk of the disease or the probability of developing the disease during the specified time period and, therefore, can be useful for etiological research. To the authors’ knowledge, however, no studies examined both the prevalence and the cumulative incidence of ASDs in a total population sample using rigorous case ascertainment since Diagnostic and Statistical Manual of Mental Disorders, 5th edition (DSM-5) was published in 2013.

The transition to DSM-5 is important in ASD. First, former diagnoses in DSM 4th edition, text revision, including autistic disorder, Asperger disorder, and pervasive developmental disorder—not otherwise specified, based on empirical studies, are now included under a single diagnosis: ASD. Additionally, DSM-IV, the prior diagnostic manual, had specified that ASD is an exclusion criterion for other neurodevelopmental disorders (NDDs), including attention deficit hyperactivity disorder (ADHD) and developmental coordination disorder (DCD). This rendered challenges not only for clinical care of individuals with ASD but also for research on phenotype and etiology among various NDDs.

The Hirosaki Five-year-old Children Developmental Health Check-up (HFC) study was established in 2013, using a total population sample of 5-year-old children residing in Hirosaki City in Japan. The rationale for the study arose from concerns about the limited data or follow-up for children who screened positive for NDDs at 18-month- or 36-month-old checkups (see Supplemental Data for more information on these checkups in Japan), despite a high level of participation in these assessments (18 months = 94.9%; 36 months = 92.9%) [10]. There is also a concern about whether the screen-positive children subsequently undergo the assessment and receive appropriate services. The present paper uses HFC data from the years 2013–2016 to estimate the prevalence and the cumulative incidence of ASDs and to examine the patterns of co-occurring NDDs, including ADHD, DCD, and intellectual disability (ID) in children with ASD in a total population sample of 5-year-old children in the Hirosaki community. With these birth cohort data from children participating in this study, we estimated the prevalence and the 5-year cumulative incidence of ASDs for each study year from 2013 until 2016. We hypothesized that the 5-year cumulative incidence of ASDs for each study year is constant, and thus increase in new ASD cases do not contribute to increased prevalence estimates of ASD.

## Method

### Study setting and participants

The present study was conducted between 2013 and 2016 in Hirosaki, a city located in Aomori, the northernmost prefecture in Japan. The city is about 524.20 km^2^ in size, with a total population of 174,171 in 71,823 households [11]. Non-Japanese residents are less than 0.5% of the total population in Hirosaki. The HFC study targeted all 5-year-old children in Hirosaki.

### Study design

Between January 2013 and December 2016, the 5-year-old developmental check-up was held annually, producing 4 cohorts. The HFC was conducted in two stages with the goal of detecting all children with NDDs in the entire city.

**Fig. 1:**
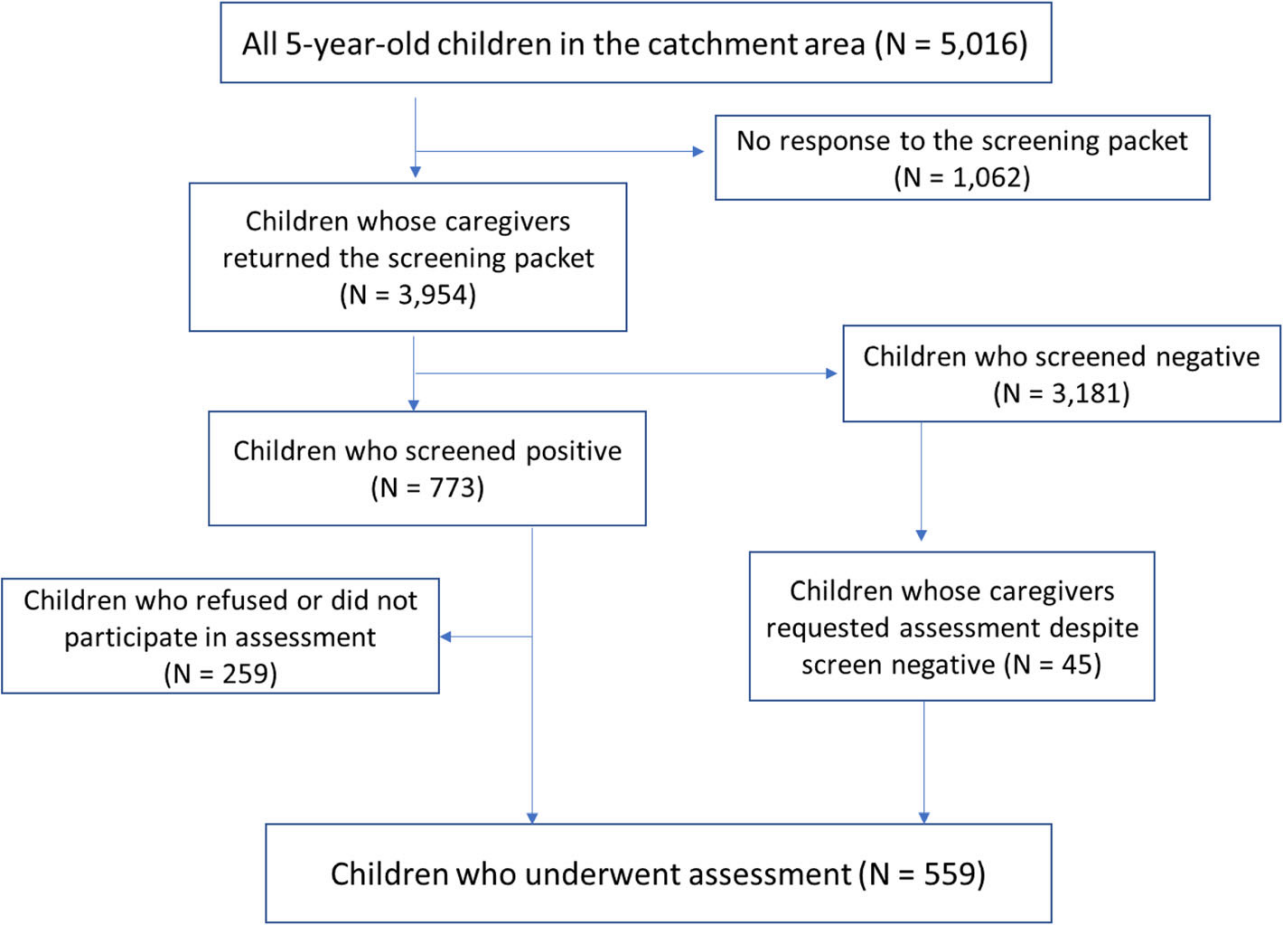
Flow chart of the Hirosaki Five-year-old Developmental Checkup and Assessment

The initial screening consisted of a set of questionnaires that the municipal health center mailed to the caregivers and kindergarten or nursery school teachers of all 5-year-old children in Hirosaki. The questionnaires included demographic information (child’s sex, family status, household income, for example) and questionnaires measuring the child’s social-emotional-behavioral challenges, as well as caregiver’s challenges that have been validated in Japanese populations. The second stage of the assessment was a comprehensive evaluation of NDDs for screen-positive children: caregivers of screen-positive children were invited to participate in a comprehensive assessment conducted at the Hirosaki University Clinic. The comprehensive assessment included child and parent interviews along with cognitive and motor skills assessments. The clinical data were taken together to arrive at specific NDD diagnoses, specifically ASD, ADHD, DCD, and ID. Figure 1 presents a flowchart of the screening and assessment stages of the HFC study.

### Initial screening stage

The initial screening questionnaires covered a wide range of children’s social-emotional-behavioral challenges and motor skills development. The following measures were used (see Supplemental Data for details): Autism Spectrum Screening Questionnaire (ASSQ), Strengths and Difficulties Questionnaire (SDQ), ADHD-Rating Scale-IV (ADHD-RS-IV), Developmental Coordination Disorder Questionnaire (DCDQ), and Parenting Stress Index (PSI) [12].

Parents completed all screeners above, and the teachers completed the SDQ. All tools above were translated to Japanese; reliability and validity were previously established [13–17] (Supplemental Data).

Children were considered “screen positive” if one of the following criteria (a)–(d) was met:

- Parent-rated ASSQ scores > 19
- PSI scores > 75 percentile of PSI score distributions
- Parent-rated ASSQ scores between 9 and 19 and parent-rated ADHD-RS total scores or at least one of subscale scores were above-defined cutoff
- One or more among parent-rated ASSQ, ADHD-RS, and DCDQ scores were above the accepted clinical cutoff and the teacher-rated SDQ total or one SDQ subscale scores were above the cutoff.

Details related to cutoff scores for each screening tool and screening criteria in the HFC study are summarized in Supplemental Data.

### Comprehensive assessment stage

All children who screened positive were invited to participate in a comprehensive assessment conducted at the Hirosaki University Clinic. Children who screened negative also took part in the assessment if their caregivers requested an evaluation.

At the comprehensive assessment, developmental history and concerns were collected using items derived from the Diagnostic Interview for Social and Communication Disorders (DISCO) [18]; the DISCO was administered by a child psychiatrist who completed a DISCO training course and/or graduate students who were trained and supervised by the child psychiatrist. The DISCO is a semi-structured interview schedule designed to collect information on development and behavior. It can be used to assist in identifying possible diagnostic categories, including ASDs and other developmental disorders affecting social interaction and communication. All of parent and child interviews were videotaped for review, case discussion, and determination of assessment fidelity. Cognitive assessments were conducted by psychologists using the Japanese version of the Wechsler Intelligence Scale for Children, 4th edition (WISC-IV) [19] only for children who did not have an ID diagnosis before participating in the present study. For children with a previously established ID diagnosis, the disability certificate was examined as a reference, and research staff confirmed that their intelligence quotient (IQ) was below 70.

To assess children’s motor skills, the Movement Assessment Battery for Children, 2nd edition (MABC-2) [20] was conducted by licensed occupational therapists and psychologists. The MABC-2 is designed to assess motor impairments of children in children aged 3–16 years, comprising 8 tasks: 3 measure manual dexterity, 2 measure ball skills, and 3 measure balance.

For cases in which high suspicions for ASD were raised by the clinical and research team after reviewing scores of the screening tools, parent and child interviews, and other testing results, children were administered the Autism Diagnostic Observation Schedule [21], by the research staff with the established ADOS research reliability.

### Case ascertainment

Each case was discussed in a multidisciplinary team that included child and adolescent psychiatrists, psychologists, a pediatrician, and occupational therapists. Best estimate diagnosis was determined based on findings from screening tools and diagnostic assessment. DSM-5 criteria were used for the diagnosis of ASD, ADHD, and DCD, and both DSM-5 and the guidelines from the European Academy of Childhood Disability [22] were used for the diagnosis of DCD. ID diagnoses were based on an IQ below 70.

### Statistical methods

For crude prevalence estimates of ASDs for each study year between 2013 and 2016, the denominator in the present study was the total number of children in the target population at the time the study was conducted each year, and the numerator was the total number of children who were diagnosed with ASDs each study year. Prevalence of ASDs for the total study years between 2013 and 2016 was computed by dividing a sum of the number of children diagnosed with ASDs each study year by a sum of the number of all 5-year-old children in the catchment area during the 4-year study period.

Adjusted prevalence of ASDs was computed using the following steps: first, the ascertained sample was divided into two groups prior to analyses:

- “High-Risk Group” (HRG): these children were identified as having disrupted development by virtue of previous clinical contact, including evaluations and receipt of early intervention services (e.g., speech therapy and occupational therapy) before the ascertainment into the HFC.
- “Low-Risk Group” (LRG): these children were never the focus of concern about developmental delays as indicated by the lack of previous evaluations or receipt of early intervention services.

Prevalence estimates were computed for each group and then combined as a single estimate. Second, for missing data on the history of service use information, values were imputed by using the distribution of this variable from available. Third, a latent variable for parent willingness to come for comprehensive evaluation was developed from patient characteristics and was tested for an association with ASD diagnosis. As ASD diagnosis was not significantly associated with the latent variable, we inferred that the likelihood of an ASD diagnosis among those who were not evaluated was not different from those who were evaluated and applied the ASD prevalence from the evaluated cases to the cases that were not evaluated. The model to predict the latent variable for parent willingness only used those who participated in the second phase. The calculated number of ASD cases was then divided by the total sample, including all children who screened negative, to compute the adjusted prevalence (see Supplemental Data for a detailed analytic plan).

We compared the phenotype characteristics of individuals who received ASD diagnoses between these HRG and LRG. The chi-square test was conducted to compare the frequency of co-existing NDDs.

For the 5-year cumulative incidence of ASD for each study year, the number of ASD cases who were born in the study catchment area each study year was divided by the total number of the birth cohort in the catchment area each study year, obtained from the municipal database [23].

The SAS 9.4 and the SPSS (Version 25.0) were used for statistical analyses.

## Results

Between 2013 and 2016, the 5016 children born in Hirosaki were eligible for the study. Caregivers of 3954 children (78.8% participation rate) completed and returned the screening packet (ASSQ, ADHD-RS, DCDQ, SDQ, and PSI). Preschool teachers completed the teacher-rated SDQ for all the 3954 children. Table 1 summarized the demographic characteristics of participating children and their families. Detailed information about participants by each study year is available in Supplemental Data. The demographic data, such as sex distribution of children and household income did not substantially differ from national statistics or those reported in a prior large Japanese epidemiological study [24, 25].

**Table 1:** Demographic data of children who returned screening questionnaire between years 2013 and 2016

	Total (***N*** = 3954)			
	***N***	%	Mean	SD
**Sex**				
Boys	2022	51.1		
Girls	1932	48.9		
**Age (months)**			62.7	3.2
**Gestational age (weeks)**				
< 28	26	0.7		
28 < = < 37	397	10.0		
37 < = < 42	3388	85.7		
42 < =	143	3.6		
**Birth weight (g)**				
< 1000	12	0.3		
1000 < = < 1500	16	0.4		
1500 < = < 2500	349	8.8		
2500 < = < 4000	3531	89.3		
4000 < =	46	1.2		
**Family structure**				
living with parents	3532	89.3		
living with a single parent	376	9.5		
Others	46	1.2		
**History of developmental abnormalities**	182	4.6		
**Familial annual income**				
< JPY 2 million	312	7.9		
JPY 2–4 million	1242	31.4		
JPY 4–7 million	1453	36.7		
JPY 7–10 million	434	11.0		
JPY 10 million < =	201	5.1		

*JPY* Japanese yen

Among children who returned the screening packet, 773 children (19.5%) were screen-positive and invited to the comprehensive assessment. Of these screen-positive children, caregivers of 259 children either refused to consent or did not bring their children for the assessment at the University Clinic, despite having given their consent. For screen-negative children (*N* = 3,181), caregivers of 45 children requested the assessment. Therefore, 559 children underwent the comprehensive in-person assessment.

Of the 559 children, 87 children (60 boys and 27 girls) were confirmed to have ASDs, yielding the crude ASD prevalence for all study years of 1.73% with 95% CI of 1.37–2.10%. Sex distribution (male to female) of the crude prevalence of ASDs was approximately 2.2:1 for the total study years. The sex-specific crude prevalence estimates of ASDs were 2.35% (95% CI 1.76–2.94%) in boys and 1.09% (95% CI 0.68–1.51%) in girls. Only 21 of these 87 children had received a diagnosis of ASD prior to the present study.

After the adjustment for non-participants in comprehensive developmental assessment, the overall prevalence of ASDs was estimated at 3.22% with 95% CI of 2.66–3.76%. The adjusted prevalence of ASDs was 4.06% (95% CI 3.20–4.92%) for boys and 2.22% (95% CI 1.57–2.88%) for girls.

The cumulative incidence of ASDs up to 5 years of age for the entire study period was 1.31% (95% CI 1.00–1.62%). The 5-year cumulative incidence for each study year is summarized in Table 2. Although the 5-year cumulative incidence increased from the year 2013 to the year 2014 and the year 2014 to the year 2015, further analysis, using a generalized linear model, revealed that there were no significant linear trends in 5-year cumulative incidence over the study years 2013–2016 (Supplemental Data).

**Table 2:** Prevalence and cumulative incidence of autism spectrum disorders for each study year and for the total study years

		Year 2013	2014	2015	2016	Total
Ascertained cases	male	14	13	18	15	60
	female	8	7	7	5	27
	total	22	20	25	20	87
*N* of children with ASD born in the catchment area	male	8	9	14	13	44
	female	5	7	6	5	23
	total	13	16	20	18	67
A total *N* of 5-year-old children in the catchment area	male	685	639	600	625	2549
	female	625	622	621	599	2467
	total	1310	1261	1221	1224	5016
*N* of 5-year-old whose caregivers returned the screening questionnaire	male	506	496	489	531	2022
	female	449	468	515	500	1932
	total	954	964	1004	1031	3954
*N* of the birth cohort in the catchment area	male	703	648	639	603	2593
	female	656	610	664	589	2519
	total	1359	1258	1303	1192	5112
Crude orevalence (%) (95% confidence interval: CI)	male	2.04 (0.98–3.10)	2.03 (0.94–3.13)	3.00 (1.64–4.36)	2.82 (1.42–4.23)	2.35 (1.76–2.94)
	female	1.28 (0.40–2.16)	1.13 (0.30–1.95)	1.13 (0.30–1.96)	0.83 (0.11–1.56)	1.09 (0.68–1.51)
	total	1.68 (0.98–2.38)	1.59 (0.90–2.28)	2.05 (1.25–2.84)	1.63 (0.92–2.34)	1.73 (1.37–2.10)
Adjusted prevalence (%) (95% CI)	male	–	–	–	–	4.06 (3.20–4.92)
	female	–	–	–	–	2.22 (1.57–2.88)
	total	–	–	–	–	3.22 (2.66–3.76)
Cumulative incidence up to 5 years of age (%) (95% CI)	male	1.14 (0.35–1.92)	1.39 (0.49–2.29)	2.19 (1.06–3.33)	2.16 (1.00–3.32)	1.70 (1.20–2.19)
	female	0.76 (0.10–1.43)	1.15 (0.30–1.99)	0.90 (0.18–1.62)	0.85 (0.11–1.59)	0.91 (0.54–1.28)
	total	0.96 (0.44–1.47)	1.27 (0.65–1.89)	1.53 (0.87–2.20)	1.51 (0.82–2.20)	1.31 (1.00–1.62)

*ASD* autism spectrum disorder, *Prevalence (%) N* of ASD cases/the total *N* of target population in the catchment area × 100

*Five-year cumulative incidence (%) N* of ASD cases who were born in the catchment area/*N* of the birth cohort in the catchment area × 100

Of all children with ASD (*N* = 87), 88.5% (*n* = 77) of children were found to have at least one co-occurring NDD (i.e., one or more among ADHD, DCD, ID, and/or borderline intellectual functioning). Of note, 20 cases (23%) had 3 co-occurring NDDs. Common co-occurring conditions include ADHD 50.6% (male:female = 2.4:1), DCD 63.2 % (male:female = 2.1:1), 36.8% ID (male:female = 1.7:1), and 20.7% borderline intellectual functioning (male:female = 2.6:1) (See Table 3).

**Table 3:** Co-occurring patterns of neurodevelopmental disorders (NDDs) in 87 individuals with autism spectrum disorder (ASD)

**Co-occurring NDDs**	***n***	**%**
None (i.e., ASD alone)	10	11.49
ADHD alone or ADHD and other NDDs	44	50.57
DCD alone or DCD and other NDDs	55	63.22
ID alone or ID and other NDDs	32	36.78
BIF alone or BIF and other NDDs	18	20.69
**Number of co-occurring NDDs**	***n***	**%**
0	10	11.49
1	25	28.74
2	32	36.78
3	29	22.99

*Totals may not add up because each pattern can overlap

(for example, individuals with ASD, ADHD, and DCD can be included in "ADHD alone or ADHD and other NDDS" and "DCD alone or DCD and other NDDS").

*ADHD* attention deficit hyperactivity disorder, *ASD* autism spectrum disorder, *BIF* borderline intellectual functioning (defined as IQ between 70 and 85 in this study), *DCD* developmental coordination disorder, *ID* intellectual disability, *NDD* neurodevelopmental disorder

Table 4 summarized comorbidity characteristics of the children in HRG (*N* = 118) and LRG (*N* = 441) who completed the comprehensive assessment. While the rate of co-occurring ID was significantly higher in children with ASD in HRG than that in children with ASD in LRG (47.5% vs. 14.3%, respectively; *p* = 0.003), rates of co-occurring ADHD and DCD were comparable for children with ASD in both HRG and LRG (55.9% vs. 39.3%, respectively; *p* = 0.15 for ADHD, 67.8% vs. 53.6%, respectively; *p* = 0.20 for DCD).

**Table 4:** Characteristics of children who underwent diagnostic assessment (*N* = 559)

	^**a**^High-risk (HR)			^**b**^Low-risk (LR)			HR + LR
	ASD	No ASD	Total	ASD	No ASD	Total	
	(*N* = 59)	(*N* = 59)	(*N* = 118)	(*N* = 28)	(*N* = 413)	(*N* = 441)	(*N* = 559)
	*N* (%)	*N* (%)	*N* (%)	*N* (%)	*N* (%)	*N* (%)	*N*
Male	38 (64.4)	42 (71,2)	80 (67.8)	22 (78.6)	223 (54.0)	245 (55.6)	325 (58.1)
ADHD	33 (55.9)	16 (27.1)	49 (41.5)	11 (39.3)	108 (26.2)	119 (27.0)	168 (30.1)
DCD	40 (67.8)	34 (57.6)	74 (62.7)	15 (53.6)	120 (29.1)	135 (30.6)	209 (37.4)
ID	28 (47.5)	15 (25.4)	43 (36.4)	4 (14.3)	22 (5.3)	26 (5.9)	69 (12.3)
BIF	9 (15.3)	14 (23.7)	23 (19.5)	9 (32.1)	67 (16.2)	76 (17.2)	99 (17.7)

*ADHD* attention deficit hyperactivity disorder, *ASD* autism spectrum disorder, *BIF* borderline intellectual functioning, *DCD* developmental coordination disorder, *ID* intellectual disability

^a^High-risk refers to children who had a history of developmental interventions prior to the study year

^b^Low-risk refers to children without any these interventions prior to the study year

## Discussion

This sequential study design allowed us to determine ASD prevalence estimates over 4 years (2013–2016) in a total population sample from Japan. In each year, all 5-year-old children in Hirosaki, Japan, were surveyed. We employed rigorous case ascertainment processes, including initial screening and subsequent comprehensive assessment that were used in the present study. Our prevalence estimate was higher than the estimated of other epidemiological studies in Asia [3, 5]. However, a significant portion of our 95% CI overlaps with that of Kim et al’s report (1.91–3.37%) [5]. Thus, observed differences in prevalence estimates in two studies could have stemmed from a few methodological differences (choice of assessment batteries and sample size, for example).

The number of male and female children diagnosed with ASD in the present study was 60 and 27, respectively, yielding the M: F ratio of 2.2 in the total study population after adjusting non-participants. While this ratio is comparable to the findings in the recent NSCH survey [6], it is lower than that reported by a recent meta-analysis of extant studies (M;F ratio of 3.25:1 [95% CI 2.93–3.62]) [26]. One possibility of accounting for such discrepancy is the differences in study populations. Compared to the studies including HRG [27, 28] alone, these studies (i.e., present and the NSCH studies) including both HRG and LRG tend to report lower male to female ratios, suggesting that researchers studying individuals with ASD identified via clinical and/or educational services might have missed females with ASD.

In the present study, participating children were divided into two groups (HRG and LRG), based on the prior use of services/interventions. Among 87 children whose ASD diagnosis was established in the present study, 28 children were from LRG, comprising approximately 30% of all children with ASD. In other words, 30% of children with ASD had not been identified as developmentally challenged and have not received any service/interventions until 5 years of age. For early identification and interventions, current findings emphasize the importance of continued follow-up developmental check-ups of children after 3 years of age.

The comparisons of annual 5-year cumulative ASD incidence allowed us to examine whether there was a true increase in ascertained ASD cases over the 4-year study period. As hypothesized, no significant changes in cumulative incidence of ASD have been identified in the study catchment area. Previously, an increase in the prevalence of ASD was considered attributable to (1) better ascertainment, (2) broadened ASD diagnostic criteria, and/or (3) a true rise in ASD incidence [29]. In the present study, the identical methods have been used to screen and diagnose children during the study period, minimizing the impact of methods variations. Our findings indicated that cumulative incidence of ASD has been stable during the 4-year study period. Using rigorous epidemiological methods to identify cases (ASD in this study) in a target population that includes all individuals at risk of developing conditions (all children born and/or live in a catchment area in this study) over significant periods (4 years in this study), our findings suggest that ASD is a common childhood-onset NDD with stable prevalence and incidence estimates.

Our study revealed high rates of co-occurring NDDs. The rate of co-occurring ID is comparable to the findings from extant research [30], whereas the rates of co-occurring ADHD and DCD were higher than those reported in a Swedish studies (36% vs. 51% and 37% vs. 63%, respectively) [30, 31]. These differences have come from differences in the methods of case ascertainment between two studies. For example, in our study, motor function in children was assessed using a battery specifically targeted to assess the movement, while it was determined by the parental interview in the Swedish study. Additionally, our study included individuals with ASD from both HRG and LRG, whereas Swedish studies included individuals only from HRG. High rates of co-occurring conditions in children with ASD in our study emphasize the necessity to evaluate co-occurring NDDs when assessing children with ASD. Given the paucity of available information in this area, it is particularly important and relevant with the advance of DSM-5.

## Limitations and strengths

The present study has several strengths: examining both ASD prevalence and the cumulative incidence, using a rigorous ascertainment process, use of DSM-5 criteria in all four years, high retention (approximately 80%) of 5-year-old children at the screening stage, sequential study design allowing us to compare the annual 5-year cumulative ASD incidence, and reporting co-occurring NDD patterns in children with ASD in a total population sample, all of which support the robustness of study findings.

Despite the strength above, findings from the present study need to be interpreted with caution, due to the following limitations: first, the present study had a modest sample size for a total population epidemiologic survey when compared to prior studies [5, 32]. This likely affects the precision of prevalence and incidence estimates as well as the confidence interval of the disease proportion [30]. Second, the ADOS data, a part of gold-standard standardized diagnostic assessments for ASD, were not available for all children because some parents declined to the ADOS testing (34 out of 87). While ASD is ultimately a clinical diagnosis, this variation in comprehensive assessment procedures might contribute to differences in ASD prevalence in the present study. Third, the model to predict the latent variable for parent willingness only used those who participated in the second phase and was based on a few variables, so it is still possible that those who were not evaluated have a different prevalence rate. Fourth, we were unable to count the exact number of children who moved out of Hirosaki city prior to the 5-year-old developmental checkup in calculating 5-year cumulative incidence, leading to biased incidence rates toward underestimation and the cumulative incidences toward overestimation [33]. However, the annual census data reporting the number of children aged 0–4 years from 2015 to 2019 revealed the number of these children who moved out of the study catchment area before age 5 was relatively steady each year and considered minimal (see Supplemental Data). Lastly, findings from the present study may not be generalizable to other communities in which population characteristics are very different such as great ancestral, social, cultural, and economic diversity. However, our findings from this Asian population are important given the limited number of epidemiologically sound studies in ASD has been conducted in this region [3–5]. Although there are still differences in ethnicity, economics, and cultures that may impact how ASD is identified and diagnosed even within this region, the accumulation of epidemiological findings would be valuable.

## Conclusions

With the use of the same ascertainment methods, including screening and diagnostic tools as well as diagnostic criteria over the four study years, our study indicated that there was no evidence of a true rise in ASD prevalence and incidence over the years 2013–2016 in our catchment area. These findings lead to the conclusion that ASD is a common childhood-onset NDD with stable prevalence and incidence estimates. Replications with larger samples and in different populations are warranted in future research.

Additionally, our study demonstrated the high frequency of co-existing NDDs (namely, ADHD, DCD, and ID) in children with ASD. This finding can support the clinical importance to provide a broad range of developmental assessments rather than ASD-focused ones for children with developmental concerns. Longitudinal studies are required to examine how the existence of co-occurring NDDs impacts long-term outcomes, such as adaptive skills, psychopathology, and well-being, in individuals with ASD.

## Supplementary information

**Additional file 1: Supplemental Data 1.** The 18-month and 36-month developmental checkups in Japan. **Supplemental Data 2.** Screening tools. **Supplemental Data 3.** Screening criteria in the Hirosaki Five-year-old Study. **Supplemental Data 4.** Detailed demographic information about study participants. **Supplemental Data 5.** Statistical methods for computing the adjusted prevalence for ASD. **Supplemental Data 6a.** Trend of 5-year cumulative incidence of ASD from the year 2013 - the year 2016. **Supplemental Data 6b.** Trend in Cumulative Incidence of ASD from 2013-2016. **Supplemental Data 7.** The census data reporting the number of children who moved out of Hirosaki city from 2015 – 2019.

## Data Availability

The datasets used during the current study are available from the corresponding author on reasonable request.

## Abbreviations

ASD: Autism spectrum disorder
ADHD: Attention deficit hyperactivity disorder
ADHD-RS: Attention deficit hyperactivity disorder-rating scale
ADOS: Autism diagnostic observation schedule
ASSQ: Autism Spectrum Screening Questionnaire
DCDQ: Developmental Coordination Disorder Questionnaire
DISCO: Diagnostic interview for social and communication disorders
HFC: Hirosaki five-year-old children developmental health check-up
HRG: High-risk group
ID: Intellectual disability
LRG: Low-risk group
NDD: Neurodevelopmental disorder
PSI: Parent stress index
SDQ: Strengths and Difficulties Questionnaire
95% CI: 95% confidence interval
