# Supplementary material for: Prevalence and cumulative incidence of autism spectrum disorders and the patterns of co-occurring neurodevelopmental disorders in a total population sample of 5-year-old children
Source: Mol Autism. 2020 May 14;11:35. doi: 10.1186/s13229-020-00342-5 (PMC7227343; doi:10.1186/s13229-020-00342-5)
Supplement: Supplementary file 1 — Additional file 1: Supplemental Data 1. The 18-month and 36-month developmental checkups in Japan. Supplemental Data 2. Screening tools. Supplemental Data 3. Screening criteria in the Hirosaki Five-year-old Study. Supplemental Data 4. Detailed demographic information about study participants. Supplemental Data 5. Statistical methods for computing the adjusted prevalence for ASD. Supplemental Data 6a. Trend of 5-year cumulative incidence of ASD from the year 2013 - the year 2016. Supplemental Data 6b. Trend in Cumulative Incidence of ASD from 2013-2016. Supplemental Data 7. The census data reporting the number of children who moved out of Hirosaki city from 2015 – 2019. [file 13229_2020_342_MOESM1_ESM.docx]

**Supplemental Data 1: The 18-month and 36-month developmental checkups in Japan**

In Japan, the Maternal and Child Health Act requires that the municipalities provide health and developmental checkups for children at 18 months and 36 months (<http://elaws.e-gov.go.jp/search/elawsSearch/elaws_search/lsg0500/detail?lawId=340AC0000000141#49>). At these checkups, children were assessed by public health nurses and pediatricians for their physical, motor, social-emotional-behavioral, and language developments as well as for overall health (medical and dental) conditions. When screened positive at these checkups, public health nurses and/or pediatricians are encouraged (not mandated) to make referrals to specialists for further assessment if necessary and to provide caregivers of children with available local resources for early interventions, such as speech therapy. There are no legal standards as to the referral systems and the number of such local resources in Japan. Following these checkups, there are no further mandatory developmental checkups provided prior to the compulsory school entry in Japan. The Ministry of Health, Labor and Welfare reports published in 2015 revealed that the participation rates in 18-month and 36-month developmental checkups in Japan were 94.9% and 92.9%, respectively. The participation rates at these checkups in the present study (years 2013 – 2016) were 98.5% and 97.7%, respectively.

**Supplemental Data 2: Screening tools**

Several questionnaires were used in the present study with the aim to identify as many children with developmental concerns as possible and refer them to diagnostic assessment. These questionnaires were chosen primarily due to financial restraints for the present study. To target a total population sample (i.e. all 5-year-old children in Hirosaki city), we chose internationally validated questionnaires below that were available at no cost. These questionnaires had undergone translation, back-translation, and standardization processes for use in the Japanese population.

1. Autism Spectrum Screening Questionnaire (ASSQ): The ASSQ is a screening tool targeting school-age children for ASD, comprising 27 items rated on a 3-point scale (1). Of 27 items, 11 items embrace social interaction, 6 items cover communication problems, and 5 items refer to restricted and repetitive behavior. The remaining items pertain to motor clumsiness and other associated symptoms. The ASSQ was translated into many languages, including Japanese (2, 3) and its psychometrics was examined in general population sample. In the present study, we set two different cut-off scores for screen-positive criteria; 1) parent-rating ASSQ total scores were above 19, which was in the top 3^rd^ percentile of the ASSQ score distributions and 2) parent-rating ASSQ total scores were between 9 and 19. The cut-off score of 19 in the first criteria was determined based on the study conducted in clinical populations in Japan, where the cut-off score of parent-rating ASSQ above 19 revealed good sensitivity (90%) and moderate specificity (67%) (4). Given that the present study included both the low-risk group (i.e. children without prior use of early interventions) and the high-risk group (i.e. children with prior use of early interventions), we also set the ASSQ score above 9 as the second cut off criteria, where approximately the top 10^th^ percentile of the AASQ scores were distributed in the present study. Children with the ASSQ score above 9 and below 19 were considered screen positive only when they concurrently had scores above cut off on the parent-rating ADHD-Rating Scale-IV or the teacher-rating Strength and Difficulties Questionnaire (see the Table below). Although the original ASSQ was standardized for children 7 – 16 years of age, using the same sample who participated in the present study, the Japanese ASSQ showed good psychometric properties in 5-year-old children (5). Details on screen-positive criteria are discussed elsewhere in this article.

2. The Strengths and Difficulties Questionnaire (SDQ): The SDQ consists of 25 items (5 scales of 5 items each), generating scores for conduct problems, hyperactivity, emotional symptoms, peer problems and prosocial behavior (6). The SDQ is widely used in both general and clinical populations to identify psychopathology as well as specific psychiatric disorders, including ASD and ADHD (7). The Japanese-version of SDQ shows comparable psychometrics to the original SDQ (8). In the present study, we used teacher-rated total difficulties score as one of the screen-positive criteria, cutoff scores of which were set as 18 for boys and 13 for girls, which captured 4.5% of boys and 5.5% of girls according to the normative data of the teacher SDQ for Japanese children aged 4-5 years (9).

3. ADHD-Rating Scale-IV (ADHD-RS-IV): The ADHD-RS-IV was developed to measure two features of ADHD: inattention (9 items) and hyperactivity-impulsivity (9 items) (10). The Japanese-version of ADHD-RS-IV was validated in a general population sample (11). Consistent with the above study conducted in Japan, the 90th percentile of the distribution of the parent-rated ADHD-RS-IV total scores was defined as the cut-off value, resulting in scores of 19 in boys and 14 in girls in the HFC study.

4. Developmental Coordination Disorder Questionnaire (DCDQ): The DCDQ is a 15-item parent questionnaire, designed to screen for coordination disorders in children aged 5–15 years (12). It was translated into Japanese, and the Japanese-version of DCDQ showed good psychometrics (13). Given limited data in the Japanese sample, we used the same cut-off scores with ones used in the study conducted by a developer of the DCDQ, defined as the15th percentile of the total DCDQ distribution in the normed sample (≤ 36 in boys and ≤ 40 in girls) (14).

5. Parenting Stress Index (PSI): The PSI is a self-report questionnaire that evaluates parenting stress over two domains: the child domain (PSI-C) and the parent domain (15). In this study, the 75th percentile level of PSI-C of the PSI-C distribution in the study sample was defined as the cut-off value based on the manual of the Japanese-version (16).

| **Supplemental Data 3: Screening criteria in the Hirosaki Five-year-old Study** | | | | | |  |  |  |
| --- | --- | --- | --- | --- | --- | --- | --- | --- |
|  |  |  |  |  |  |  |  |  |
| \| Cut-off scores \| \| \| \| \| \| \| \| \|  \| \| --- \| --- \| --- \| --- \| --- \| --- \| --- \| --- \| --- \| --- \| \|  \|  \| Male-specific \| Female-specific \|  \| Criteria 1 \| Criteria 2 \| Criteria 3* \| Criteria 4** \|  \| \| ASSQ \| 19 \|  \|  \|  \|  \|  \|  \|  \|  \| \| ASSQ \| 9 \|  \|  \|  \|  \|  \| a \| c \|  \| \| ADHD-RS: Inattention \|  \| 12 \| 9 \|  \|  \|  \| d \|  \| \| ADHD-RS: Hyperactivity/Impulsivity \|  \| 8 \| 5 \|  \|  \|  \|  \| \| ADHD-RS: Total \|  \| 19 \| 14 \|  \|  \|  \|  \| \| DCDQ-R: Control during movement \|  \| 14 \| 14.3 \|  \|  \|  \|  \|  \| \| DCDQ-R: Fine motor \|  \| 8 \| 10.7 \|  \|  \|  \|  \|  \| \| DCDQ-R: General coordination \|  \| 11 \| 13 \|  \|  \|  \|  \|  \| \| DCDQ-R: Total \|  \| 36 \| 40 \|  \|  \|  \|  \|  \| \| PSI \| 75 percentile \|  \|  \|  \|  \|  \|  \|  \|  \| \| SDQ-T Total \|  \| 18 \| 13 \|  \|  \|  \| b \|  \|  \| \|  \|  \|  \|  \|  \|  \|  \|  \|  \|  \| \| * Criteria 3 was met if "SDQ-T Total score was above cut-off (b)" AND "at least one of the following parent-rating scales (either total score or sub-scale score) was above cut-off (a)" \| \| \| \| \| \| \| \| \| \| \| ** Criteria 4 was met if "ASSQ score was above 9 (c)" AND "two or more among ADHD-RS total score or subscale score was above cut-off (d)" \| \| \| \| \| \| \| \| \| \|   **Abbreviations**: ASSQ = Autism Spectrum Screening Questionnaire, ADHD- RS = Attention Deficit Hyperactivity Disorder-Rating Scale, DCDQ = Developmental Coordination Disorder Questionnaire, PSI =Parenting Stress Index, SDQ-T = Strength and Difficulties Questionnaire-Teacher form | | | | | | | | |
|  |  |  |  |  |  |  |  |  |
|  |  |  |  |  |  |  |  |  |

**Supplemental Data 4: Detailed demographic information about study participants**

|  | | | 2013 | % | 2014 | % | 2015 | % | 2016 | % | Total | % |
| --- | --- | --- | --- | --- | --- | --- | --- | --- | --- | --- | --- | --- |
| All 5-year-old Children | | | 1310 |  | 1261 |  | 1221 |  | 1224 |  | 5016 |  |
|  |  | male | 685 | 52.3 | 639 | 50.7 | 600 | 49.1 | 625 | 51.1 | 2549 | 50.8 |
|  |  | female | 625 | 47.7 | 622 | 49.3 | 621 | 50.9 | 599 | 48.9 | 2467 | 49.2 |
| Children who underwent initial screening stage | | | 954 | 72.8 | 965 | 76.5 | 1004 | 82.2 | 1031 | 84.2 | 3954 | 78.8 |
|  |  | Male | 505 | 52.9 | 495 | 51.3 | 489 | 48.7 | 531 | 51.5 | 2020 | 51.1 |
|  |  | Female | 449 | 47.1 | 470 | 48.7 | 515 | 51.3 | 500 | 48.5 | 1934 | 48.9 |
| Children who did not participate in screening | | | 356 | 27.2 | 296 | 23.5 | 217 | 17.8 | 193 | 15.8 | 1062 | 21.2 |
| Children who screened positive | | | 226 | 23.7 | 179 | 18.6 | 202 | 20.1 | 166 | 16.1 | 773 | 19.6 |
| Children who screened negative | | | 728 | 76.3 | 786 | 81.5 | 802 | 79.9 | 865 | 83.9 | 3181 | 80.5 |
| Children whose caregivers requested assessment despite being screen negative | | | 0 |  | 13 |  | 18 |  | 14 |  | 45 |  |
| Total number of children who underwent comprehensive assessment | | | 159 |  | 123 |  | 158 |  | 119 |  | 559 |  |
| Children whose caregivers refused comprehensive assessment | | | 67 |  | 69 |  | 62 |  | 61 |  | 259 |  |

**Supplemental Data 5: Statistical methods for computing the adjusted prevalence for ASD**

In the sample of children who screened positive or children whose caregivers requested an assessment despite screening negative (n=818), 67 children were missing information on the history of service use. All 67 of these children were from the group who screened positive but refused or did not participate in assessment (n=259). In this subgroup of 259 children, patterns of missing data were examined to determine if the distributions of other characteristic values (sex, ASSQ, ADHD-RS, DCDQ, and SDQ) associated with cases missing history of service use information differed from the distributions of values associated with complete cases. As the distributions were similar, the value for history of service use from the complete cases (5% had a history of service use) was imputed for the cases that were missing the history of service use. A “yes” response was randomly assigned to 5% of the case that was missing the history of service use information.

Then, in order to determine how to handle screen positive children who did not come for comprehensive evaluation (comprehensive evaluation non-participants) in estimating prevalence, it was hypothesized that there exists in each child’s parents a latent variable that cannot be directly measured but represents parental willingness to further participate in the comprehensive diagnostic process. If this latent variable is independent of ASD diagnoses, then the observed ASD diagnosis rates in the group of children completing confirmative diagnostic assessments provide a reasonable basis for a weighted estimate of prevalence in the entire screen-positive children in the study. More specifically, logistic regression was performed with parent willingness for evaluation (559 yes, 259 no) as the dependent variable and sex, history of service use, ASSQ (≥9 vs. <9), and teacher SDQ total (above vs. below cut-off) as the independent variables. A latent variable for parent willingness for evaluation was created from the predicted probability values and was then tested for an association with ASD diagnosis in a generalized linear model (GLM), controlling for the same covariates. These models were run in the overall sample (n=818) and in the following four subgroups: girls (n=375), boys (n=443), high risk (n=132; history of service use), and low risk (n=686; no history of service use). As ASD diagnosis was not significantly associated with the latent variable, we inferred that the likelihood of an ASD diagnosis among those who were not evaluated was not different from those who were evaluated and applied the ASD prevalence from the evaluated cases to the cases that were not evaluated. However, the model to predict the latent variable for parent willingness only used those who participated in the second phase and was based on a few variables, so it is still possible that those who were not evaluated have a different prevalence rate. The calculated number of ASD cases was then divided by the total sample, including all children who screened negative, to compute the adjusted prevalence.

**Supplemental Data 6a: Trend of 5-year cumulative incidence of ASD from the year 2013 - the year 2016**

**
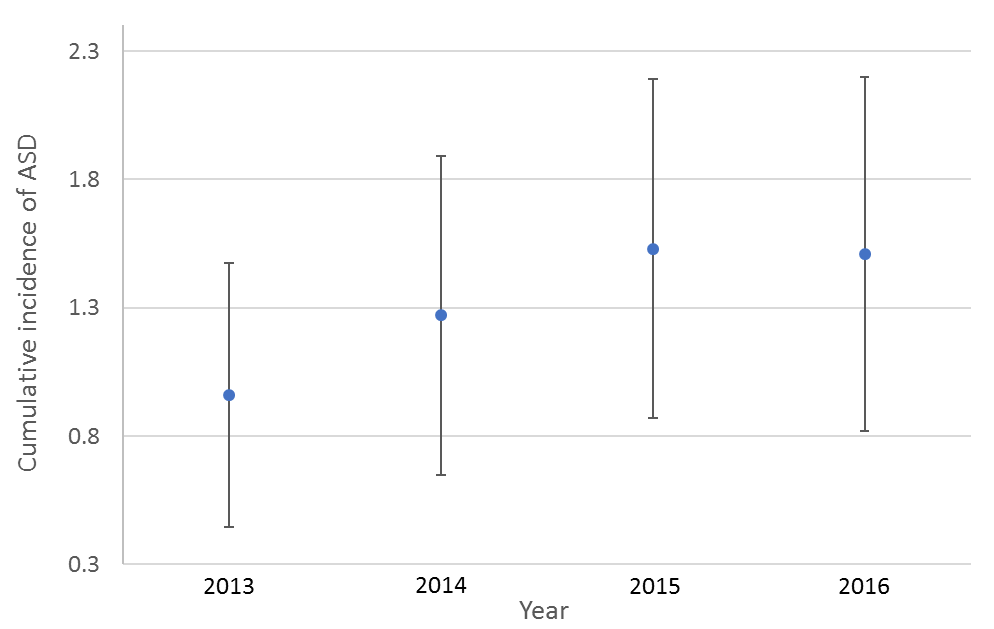
**

There was no significant linear trend identified in overall cumulative incidence of ASD from Generalized linear modeling (p = 0.07) during study years. Similarly, neither cumulative incidence trend in male nor that in female was significant (p = 0.07 and 0.98, respectively).

**Supplemental Data 6b. Trend in Cumulative Incidence of ASD from 2013-2016**

|  | **Slope from GLM** | **95% CI** | **p-value** |
| --- | --- | --- | --- |
| Overall | 0.191 | -0.044, 0.426 | 0.073 |
| Male | 0.386 | -0.075, 0.847 | 0.069 |
| Female | 0.002 | -0.392, 0.395 | 0.985 |

GLM = Generalized Linear Model, CI = confidence interval

**Supplemental Data 7. The census data reporting the number of children who moved out of Hirosaki city from 2015 – 2019**

|  |  | age | | | | |  |  |
| --- | --- | --- | --- | --- | --- | --- | --- | --- |
|  |  | 0-1 | 1-2 | 2-3 | 3-4 | 4-5 | total | Number of children aged 0 - 5 * |
| year | 2015 | 42 | 72 | 63 | 59 | 54 | 290 | 5923 |
|  | 2016 | 45 | 99 | 65 | 65 | 53 | 327 | 5928 |
|  | 2017 | 42 | 116 | 71 | 57 | 70 | 356 | 5914 |
|  | 2018 | 33 | 104 | 78 | 64 | 42 | 321 | 5781 |
|  | 2019 | 38 | 71 | 71 | 38 | 33 | 251 | 5571 |

* Number of children (aged 0 - 5) who resided in Hirosaki city at the time of the census

**References**:

1. Ehlers S, Gillberg C. The epidemiology of Asperger syndrome. A total population study. J Child Psychol Psychiatry. 1993; 34:1327–1350.

2. Ito H, Matsumoto K, Takayanagi N, Harada S, Ohtake S, Mochizuki N, et al. Psychometric properties of the Japanese version of the Autism Spectrum Screening Questionnaire (ASSQ): development of a short form [in Japanese]. Shinrigaku Kenkyu. 2014; 85:304–12.

3. Mattila M-L, Jussila K, Linna S-L, Kielinen M, Bloigu R, Kuusikko-Gauffin S, et al. Validation of the Finnish Autism Spectrum Screening Questionnaire (ASSQ) for clinical settings and total population screening. J Autism Dev Disord. 2012; 42:2162–2180.

4. National Institute of Special Needs Education. Yokosuka: Research report [cited 2018 Dec 15]. 7 p: Available from http://www.nise.go.jp/kenshuka/josa/kankobutsu/pub_f/F-112/04.pdf

5. Adachi M, Takahashi M, Takayanagi N, Yoshida S, Yasuda S, Tanaka M, et al. Adaptation of the Autism Spectrum Screening Questionnaire (ASSQ) to preschool children. PloS One. 2018; 13: e0199590.

6. Goodman R. The Strengths and Difficulties Questionnaire: a research note. J Child Psychol Psychiatry. 1997; 38:581–586.

7.Russel G, Rodgers LR, Ford T. The strengths and difficulties questionnaire as a predictor of parent-reported diagnosis of autism spectrum disorder and attention deficient hyperactivity disorder. PLoS One. 2013; 8: e80248.

8. Moriwaki, A., Kamio, Y. (2014). Normative data and psychometric properties of the strengths and difficulties questionnaire among Japanese school-aged children. Child and Adolescent Psychiatry and Mental Health. 21;8(1):1. doi: 10.1186/1753-2000-8-1.

9. Iida, Y., Moriwaki, A., Komatsu, S., Kamio, Y. (2014). Standardization of the Strengths and Difficulties Questionnaire among Japanese 4-5 year-old preschool children, In: Kamio, Y., ed. *Annual report of research supported by health and labour sciences research grants. Prevalence of developmental disorders and its developmental change: a community-based cross-sectional and prospective study.* [In Japanese], National Center of Neurology and Psychiatry, Tokyo, pp.33-41.

10. Dupaul GJ, Power TJ, McGoey KE, et al.: Reliability and validity of parent and teacher ratings of attention-deficit/hyperactivity disorder symptoms. J Psychoeduc Assess 1998; 16:55–68

11. Takayanagi N, Yoshida S, Yasuda S, Adachi M, Kaneda-Osato A, Tanaka M, et al. Psychometric properties of the Japanese ADHD-RS in preschool children. Res Dev Disabil. 2016; 55:268–278.

12. Wilson BN, Kaplan BJ, Crawford SG, Campbell A, Dewey D. Reliability and validity of a parent questionnaire on childhood motor skills. Am J Occup Ther Off Publ Am Occup Ther Assoc. 2000; 54:484–493.

13. Nakai A, Miyachi T, Okada R, Tani I, Nakajima S, Onishi M, et al. Evaluation of the Japanese version of the Developmental Coordination Disorder Questionnaire as a screening tool for clumsiness of Japanese children. Res Dev Disabil. 2011; 32:1615–1622.

14. Wilson BN, Crawford SG, Green D, Roberts G, Aylott A, Kaplan BJ. Psychometric properties of the revised Developmental Coordination Disorder Questionnaire. Phys Occup Ther Pediatr. 2009; 29:182–202.

15. Abidin R: The Parenting Stress Index (3rd ed.). Psychological Assessment Resources. 1995; Odessa, FL

16. Narama M, Kanematsu Y, Araki M, Maru M, Nakamura N, Takeda J, et al. Validity and Reliability of the Japanese Version of the Parenting Stress Index [in Japanese]. The Journal of Child Health. 1999; 58: 610-616.
